# “Motion or Emotion? Recognition of Emotional Bodily Expressions in Children With Autism Spectrum Disorder With and Without Intellectual Disability”

**DOI:** 10.3389/fpsyg.2020.00478

**Published:** 2020-03-25

**Authors:** Noemi Mazzoni, Isotta Landi, Paola Ricciardelli, Rossana Actis-Grosso, Paola Venuti

**Affiliations:** ^1^ODFLab – Department of Psychology and Cognitive Sciences, University of Trento, Rovereto, Italy; ^2^MPBA, Fondazione Bruno Kessler, Trento, Italy; ^3^Department of Psychology, University of Milano – Bicocca, Milan, Italy; ^4^Milan Centre for Neuroscience, Milan, Italy

**Keywords:** autism spectrum disorder, biological motion, emotional bodily expressions, emotion recognition, intellectual disability

## Abstract

The recognition of emotional body movement (BM) is impaired in individuals with Autistic Spectrum Disorder ASD, yet it is not clear whether the difficulty is related to the encoding of body motion, emotions, or both. Besides, BM recognition has been traditionally studied using point-light displays stimuli (PLDs) and is still underexplored in individuals with ASD and intellectual disability (ID). In the present study, we investigated the recognition of happy, fearful, and neutral BM in children with ASD with and without ID. In a non-verbal recognition task, participants were asked to recognize pure-body-motion and visible-body-form stimuli (by means of point-light displays-PLDs and full-light displays-FLDs, respectively). We found that the children with ASD were less accurate than TD children in recognizing both the emotional and neutral BM, either when presented as FLDs or PLDs. These results suggest that the difficulty in understanding the observed BM may rely on atypical processing of BM information rather than emotion. Moreover, we found that the accuracy improved with age and IQ only in children with ASD without ID, suggesting that high level of cognitive resources can mediate the acquisition of compensatory mechanisms which develop with age.

## Introduction

As social individuals, we are constantly surrounded by other people and daily involved in social interactions. Hence, the ability to comprehend correctly the social signals is fundamental to our species. Among the social signals, the body movement (BM) plays a key role in conveying other people’s feeling, intentions, and emotions and its importance is comparable to that of facial expressions (de Gelder et al., 2010; de Gelder and de Borst, 2015), or even more (Van den Stock et al., 2007; Aviezer et al., 2012).

A solely kinematic description of BM is conveyed by biological motion, traditionally represented by means of point light displays (PLDs, Johansson, 1973). In typical developing individuals (TD), such a description is sufficient for inferring a great amount of social information, such as identity (Kozlowski and Cutting, 1977; Troje et al., 2005); gender (Kozlowski and Cutting, 1977; Pollick et al., 2002; Johnson et al., 2011); the nature of the actions (Johansson, 1973; Dittrich, 1993; Alaerts et al., 2011); intentions (Roché et al., 2013); and – more importantly to the present study – emotions (Dittrich et al., 1996; Atkinson et al., 2004; Clarke et al., 2005; Roether et al., 2009).

In contrast, the ability to perceive and recognize the BM seems to be impaired in autisms spectrum disorder (ASD). ASD is a neurodevelopmental disorder characterized by persistent deficits in social communication and social interaction, in social-emotional reciprocity, and in non-verbal communicative behavior (American Psychiatric Association, 2013). In the last two decades, an increasing amount of studies have suggested that social difficulties in ASD may be associated with difficulties in processing the BM (Blake et al., 2003; Dakin and Frith, 2005; Annaz et al., 2010) and in understanding the information that it conveys (Grèzes et al., 2009; Kaiser et al., 2010; Mcpartland et al., 2012; Pavlova, 2012). For instance, children with ASD showed early difficulties in orienting toward BM (Klin and Jones, 2008; Klin et al., 2009) and no preference for biological over scrambled motion (Blake et al., 2003; Annaz et al., 2012). Moreover, difficulties in recognizing *emotional* BM has been consistently reported in children (Moore et al., 1997; Fridenson-hayo et al., 2016), adolescents (Hubert et al., 2007; Parron et al., 2008) and adults with ASD (Atkinson, 2009; Philip et al., 2010; Nackaerts et al., 2012; Alaerts et al., 2014) or with high autistic traits (Actis-Grosso et al., 2015). However, other studies failed to find differences between neurotypical individuals and people with ASD when emotionally *neutral* movements were presented (Moore et al., 1997; Hubert et al., 2007; Parron et al., 2008; Murphy et al., 2009; Saygin et al., 2010). Therefore, it is still not clear whether the social difficulty in ASD is related to difficulties in processing the BM, the emotional content, or both.

Notably, most of the previous studies have focused on PLDs recognition. However, in daily life the body form is fully visible and this should be taken into account when social difficulties are investigated. Studies that have used stimuli with visible body form (full-light display stimuli – FLDs) together with PLDs, are scarce. Research in TD children (Ross et al., 2012) and adults (Atkinson et al., 2004) found an advantage in recognizing emotional FLDs compared to PLDs, suggesting a better recognition of emotional BM when more natural – although more complex – stimuli were used. In ASD, the investigation of differences in recognizing emotional PLDs and FLDs was limited to adults and showed an impaired recognition of both PLDs and FLDs (Atkinson, 2009). Poorer accuracy in recognizing emotional FLDs of BM have been shown in children with ASD (Fridenson-hayo et al., 2016). Yet, differences in recognizing emotional FLDs and PLDs have not been investigated.

Furthermore, individuals with ASD and intellectual disabilities (ID; namely with an IQ < 70) and/or with language impairment have rarely been included in past research on emotional BM recognition. Indeed, most of the previous studies have investigated the recognition of emotional BM in people without ID (namely with an IQ ≥ 70), using verbal tasks and verbal abilities as groups matching criteria. Critically, the cognitive profile of people with ASD is commonly uneven with non-verbal abilities higher than verbal ones (e.g., Happé, 1994; Joseph et al., 2002; Black et al., 2009). Therefore, it is possible that the groups of TD and ASD participants were not well matched in past research, since similar verbal skills might correspond to higher non-verbal abilities in people with ASD. Moreover, the social skills of people with ASD vary considerably according to IQ as good cognitive abilities may mediate the acquisition of compensatory strategies – that are declarative and learned explicitly through experience and/or training – for dealing with social and emotional signals (McKay et al., 2012; Rutherford and Troje, 2012). Altogether, these issues could explain why some studies failed to find differences between people with ASD and controls (Herrington et al., 2007; Freitag et al., 2008; Murphy et al., 2009; Saygin et al., 2010). An examination of emotional BM recognition in children with ASD using non-verbal task and non-verbal IQ as group-matching criteria is thus desirable. In the absence of verbal components, such procedure could also be used to compare children with ASD with and without ID or verbal disorders.

On these premises, the aim of the present study was to investigate which are the critical aspects of emotional BM that are impaired in children with ASD (i.e., difficulty in the processing of the emotional content, the BM, or both). Building on previous studies, we presented both stimuli conveying motion-kinematic-only (PLDs) and stimuli where the body form was visible (FLDs); we included children with ASD with and without intellectual disabilities (ID); we adopted the non-verbal IQ as matching criteria; and we asked the participants to perform a non-verbal simplified emotion recognition task. To assess the role of body motion information processing and emotion recognition, we presented emotional (happy–positive; fearful-negative) and neutral BM. If the difficulty in recognizing the meaning of BM in ASD were associated with difficulties in the encoding and/or processing of body motion, we would expect an impaired recognition of neutral as well as emotional BMs. Conversely, if the difficulty in recognizing the meaning of BM were related to the processing and recognition of the emotional content, children with ASD would show a specific impairment in the recognition of happy and/or fearful, but not neutral, movements. Likewise, by comparing PLDs and FLDs, we aimed at testing whether the difficulty in children with ASD was related to the visual characteristics (i.e., pure kinematic or visible body form) of the stimuli or instead to difficulties in processing the BM *per se*. If that were the case, we would expect an impaired performance in both display types (PLDs and FLDs). By contrast, if the impairment were specifically related to a difficulties in perceiving a human being depicted as PLDs (due to the tendency of individuals with ASD to process parts of the stimuli and their difficulty to integrate these part into a gestalt, e.g., Happé and Frith, 2006), an impaired performance in PLDs, but not in FLDs, should be found. Finally, regarding the age- and the non-verbal IQ- factors, we predicted that the ability to recognize emotional BM would improve with age in TD children (Ross et al., 2012) but to a lesser extent in children with ASD (Blake et al., 2003; Annaz et al., 2010). In addition, we expected that higher cognitive resources would lead to a more accurate and faster BM recognition in TD children, and, in turn, would allow ASD children to compensate and obviate, at least in part, their impairment in BM comprehension.

## Materials and Methods

### Stimuli

Participants were presented with short video-clips displaying PLDs and FLDs of whole-body expressions of happiness, fear, and neutral actions. The stimuli were adapted from a larger set of stimuli (Atkinson et al., 2004, 2007). The FLDs consisted of 3-s digital movies depicting a gray-scale actor moving against a black background. To ensure that the BM was the only source of social signals available to the observer, the actors’ face was covered. The PLDs lasted 2 s and consisted of 13 lighting dots placed over the main joints of the actor, moving against a black background, and were created by converting the FLDs stimuli to PLDs (Atkinson et al., 2012). Examples of the stimuli can be viewed at https://atkinsonap.github.io/stimuli/. Each display type included 10 different bodily expressions of each emotion, which vary in intensity and type of movements. This variability made the stimuli representative of a broad range of bodily emotions, but has the potential problem of a different ease of recognition for the stimuli with different intensity, given that a direct correlation between emotions’ intensity and recognizability has been demonstrated in a similar set of stimuli (Dittrich et al., 1996; Atkinson et al., 2004). Thus, in a separate pilot study, we asked to 21 TD children between 5 and 11 years old (10 females and 11 males; *M*_*age*_ = 9.29; *SD*_*age*_ = 1.45) to rate the intensity of the expressed emotion by using a 9-point Likert scale. Stimuli were presented according to the intensity index obtained from this pilot study (see section “Procedure”). Specifically, the order of the video was based on the recognition ease and ranged from the easiest to the most difficult video, according to the rate established in the pilot study.

### Participants

A total of 27 TD children, 25 children with ASD without ID (HFA) and 17 children with ASD and ID (LFA) participated in this study, for a total of 69 children (Table 1). Children with ASD were recruited at the Laboratory of Observation, Diagnosis, and Education (ODFLab) – University of Trento, the Autism Parents Association in Trento (AGSAT), and the Istituto Dosso Verde in Milan. All the children with ASD met the established criteria for ASD as specified in DSM-IV (American Psychiatric Association, 2000) or DSM-5 (American Psychiatric Association, 2013). The diagnosis of ASD was made by experienced clinicians, on the basis also of the administration of the Autism Diagnostic Observation Schedule (Lord et al., 2000), or based on the Autism Diagnostic Interview (ADI/ADI-R) (Lord et al., 1994). The non-verbal IQ score was measured by Raven’s progressive matrices (Raven, 1941), the Colored progressive matrices (John and Raven, 2003), or the subscales of Leiter-R brief IQ (Roid and Miller, 1997) according to the participants’ age and cognitive difficulties. TD children were recruited from the general population via the personal network of the researchers and master students involved. Specific data on socioeconomic status were not recorded. The study was approved by the Ethical Committee of the University of Milano-Bicocca. Before the experiment, the participants and their parents received a detailed explanation of the procedure and provided a signed informed consent, in accordance with the Declaration of Helsinki.

**TABLE 1:** Descriptive statistics: numerosity (N), female:male ratio (F:M), means and standard deviations (SD) of the chronological age, mental age, and IQ are reported in the three groups of children (HFA, LFA, and TD).

**Group of functioning**	**N**	**Chronological age (years)**	**Mental age (years)**	**IQ**
	
	**(F:M)**	***Mean (SD)***	***Mean (SD)***	***Mean (SD)***
ASD	42 (3:39)			
Without ID (HFA)	25 (1:24)	9.88 (2.96)	9.77 (3.26)	100.16 (19.48)
With ID (LFA)	17 (2:15)	12.33 (2.19)	5.429 (1.76)	44.87 (14.87)
TD	27 (13:14)	8.81 (1.84)	9.69 (2.33)	110.00 (14.74)

### Procedure

**FIGURE 1:**
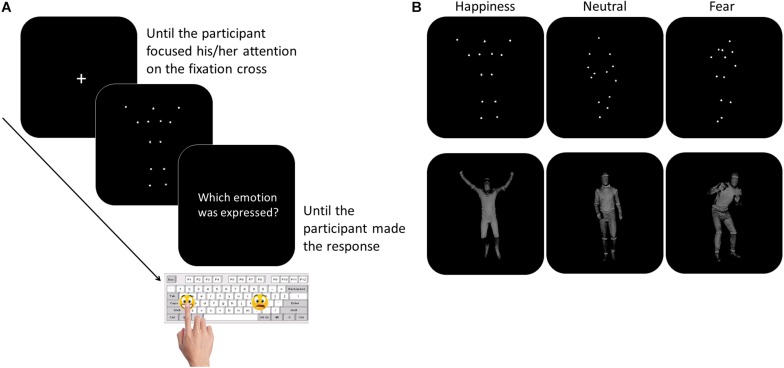
Participants performed a two forced-choice non-verbal task, during which they were asked to recognize the emotion content of whole- BMs by pressing the corresponding emoticon on the keyboard (Panel **A**). The stimuli included happy, neutral, or fearful body movements depicted as PLDs (Panel **B**, top row) and FLDs (Panel **B**, bottom row).

The participants were tested individually in a quiet room. They sat in front of a PC, at a distance of 60 centimeters from the computer monitor. The experimenter sat next to the participant for the entire duration of the study. To overcome the verbal impairment and the difficulties in focusing and in maintaining the attention often described in children with ASD (Goldstein et al., 2001; Happé et al., 2006), we asked the participants to perform a non-verbal recognition task. Moreover, to limit the task attentional and working memory load, the number and the duration of the experimental blocks were minimized, as well as the response options. Thanks to this simplified procedure, the children with LFA and language difficulties were also able to perform the task. The task consisted of a two-forced choice categorization of the emotional content of BMs, made by pressing a designated key on the computer keyboard. Sticky emoticons reproducing the facial expression corresponding to the type of emotions presented through BM was stuck on the response keys (Figure 1). At the beginning of each block, the participants performed a brief practice session. Each movie was preceded by a fixation cross, only when the child paid attention to the monitor the experimenter started the video. Each video was presented once. At the end of each video, the video disappeared and the question “Which emotion was expressed?” was presented until the participant made the response (Figure 1). The participant was asked to categorize the observed video by pressing the key with the corresponding facial emoticon, as accurately and fast as possible. Accuracy and response times (RTs) were recorded. If the participant did not recognize the emotion expressed in the video and was not able to choose between the two response options, the experimenter pressed a defined key (namely, the key corresponding to the letter “g” in the block “Fear-Happiness”; “a” in the block “Neutral-Happiness”; and “l” in the block “Fear-Neutral”) and a new video was presented. This key was different between the blocks to prevent the children from learning which was the key that would have allowed them to skip the response. Stimulus presentation was controlled and behavioral responses were recorded with E-Prime 2.0 software^®^ (Psychology Software Tools, Inc). The task was divided into three blocks, each block included videos of only two types of emotion and only two response options were possible (Fear-Happiness; Fear-Neutral; Happiness-Neutral). The order of the blocks was counterbalanced among the participants. Each block included 10 PLDs and 10 FLDs for each type of emotion, for a total of 40 videos per block (20 FLDs and 20 PLDs). Within each block, PLDs and FLDs were presented separately, half of the participants saw PLDs first and then FLDs, the other half vice versa. Within each block, for both PLDs and FLDs and for each emotion type, the order of the video was based on the recognition ease and ranged from the easiest to the most difficult video, according to the rate established in the pilot study (see section “Stimuli”). By adopting this pseudo-randomized order of presentation, we aimed at meeting the ASDs’ attentional limits, thus optimizing their performance. In fact, a complete randomized order of stimulus presentation might have resulted in two – or even more – difficult videos to be consecutively presented within the first trials of the block, inducing participants with ASD to prematurely disengage their attention from the task.

### Statistical Analysis

All the analyses were performed with the software R, package 3.3.1 (R Core Team, 2016). Wilcoxon-rank-sum test with continuity correction showed that TD children were matched with the group of children with HFA for chronological age (*W* = 297.5, *p* = 0.246), mental age (*W* = 389.5, *p* = 0.671), and IQ (*W* = 471, *p* = 0.067). The group of children with LFA was chronologically older but mentally younger than the group of TD children (chronological age: *W* = 367, *p* < 0.001; mental age: *W* = 28, *p* < 0.001) and children with HFA (chronological age: *W* = 117, *p* = 0.025; mental age: *W* = 365, *p* < 0.001). The IQ level in children with LFA was lower than the IQ in children with HFA (*W* = 405, *p* < 0.001) and TD children (*W* = 0, *p* < 0.001) (Table 1). The study sample size was empirically set at ∼25 subjects per group, namely TD, LFA, and HFA, for a total of 75 subjects. In particular, this numerosity was determined taking into account the sample size of previous studies in the field, the complexity of the task that was presented, and its feasibility for individuals with LFA. Despite being influenced by group composition, recruitment areas, and available means, the sample size selected was consistent with previous literature on the subject (Moore et al., 1997; Atkinson et al., 2004; Hubert et al., 2007; Parron et al., 2008; Atkinson, 2009; Annaz et al., 2010, 2012; Fridenson-hayo et al., 2016). Eventually, we successfully included in the study 69 subjects (27 TD; 25 HFA; 17 LFA) that completed the task. We performed a *post hoc* power analysis to check whether this sample size was acceptable for the 3 × 2 × 3 mixed ANCOVA model to detect an effect. We ran a *post hoc F*-test power analysis (R package *pwr)* with medium effect size of 0.15 and sample size equal to 69 (see estimated effect size from Federici et al., 2019). We obtained that the power of detecting such an effect at 0.05 level is equal to 0.90.

As a measure of *accuracy*, the percentages of the videos correctly categorized were calculated for each participant for each emotion type both for FLDs and PLDs. Since the mean percentages of correct responses was not normally distributed, the arcsine square root transformation of the proportions of correct responses was performed and all the following analyses were made on the transformed data.

Response times (RTs) were defined as the total amount of time (in milliseconds) from the offset of the video to the participant’s response. Only the RTs relative to correct responses were included in the analyses. For each participant, the RTs considered as outliers according to the Tukey’s method were discharged (Tukey, 1977; Hoaglin et al., 1986.; Rousseeuw and Leroy, 1987; Ratcliff, 1993; Hoaglin, 2003; Hodge et al., 2004), for a total of 22.82% of the trials (specifically 12.26% in the TD group, 25.91% in the HFA and 36.76% in the LFA). Since the mean RTs of correct responses was not normally distributed, the natural logarithms transformation of the averaged RTs was performed and all the following analyses were made on the transformed data.

Two participants with LFA were excluded from the final analysis because they did not terminate the task. Since the female:male ratio was higher in TD group than in the ASD groups, differences between TD females and males were investigated using *t*-tests. No differences were found both in Accuracy (*t* = −0.033, df = 24.79, *p*-value = 0.974) and in RTs (*t* = 1.378, df = 24.07, *p*-value = 0.181), therefore the variable “sex” was not considered in the further analysis. Analyses of both Accuracy and RTs were performed using 3 × 2 × 3 mixed ANCOVAs with Group as the between-factor (TD, HFA, and LFA); Display (FLDs, PLDs) and Emotion (Fear, Happiness, Neutral) as within-factors; non-verbal IQ and Age (chronological age) as covariates. The generalized eta squared values (η^2^_*G*_) (Olejnik and Algina, 2003) were also reported as an additional metric of effect size for all significant or marginally significant effects or interaction. Homoscedasticity of variances was tested with Levene-Test. Standard residuals were examined with Shapiro-Wilk normality test and by means of a quantile-quantile plot (QQ-plots) to check that data were normally distributed before parametric statistics were applied. *Post hoc* comparisons were performed using pairwise *t*-tests and the significance of alpha level was adjusted with Bonferroni’s correction (adjusted p-values are reported). Finally, to simultaneously investigate the trade-off between the speed and the accuracy of a response and better understand how the strategies vary among functioning groups we conducted a speed-accuracy trade-off (SATO) analysis.

## Results

### Analysis of Accuracy

#### Overall Analysis

**FIGURE 2:**
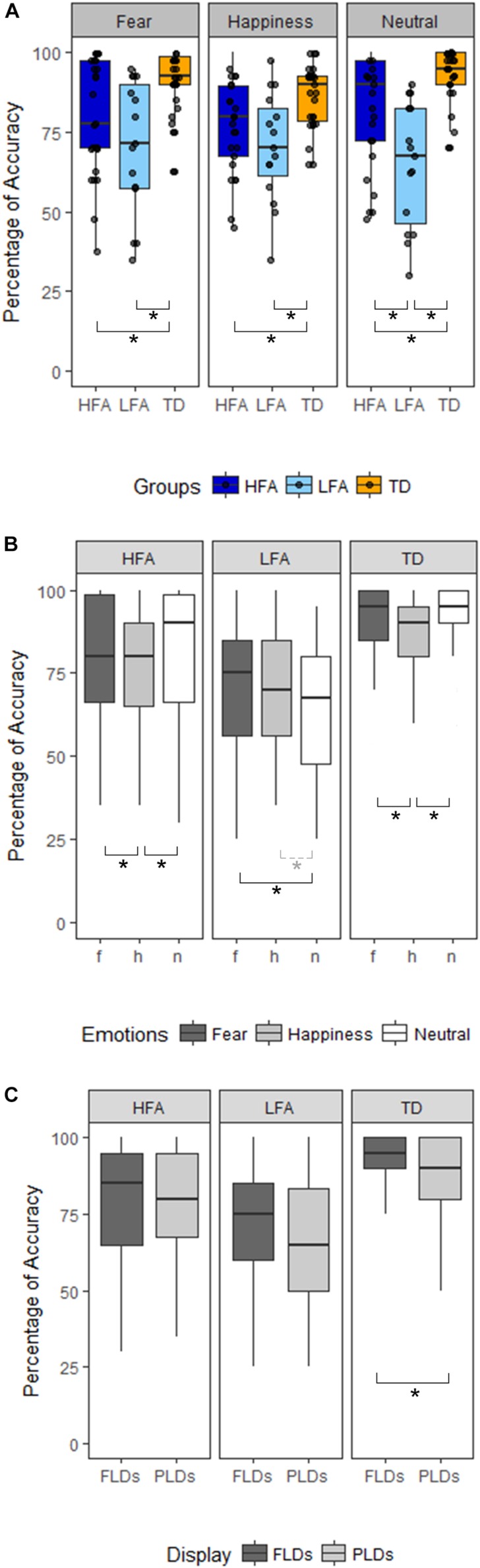
Panel**(A)** shows the differences between the three groups of children (HFA: ASD without ID, LFA: ASD with ID; TD: typical developing) in the three emotional categories (Happiness, Fear, and Neutral). The black dots represent the percentage of Accuracy of every participant in the relative emotional category. Panel **(B)** shows within-group differences between the emotional categories (dark-gray for Fear, light-gray for Happiness, white for Neutral stimuli). Panel **(C)** shows within-group differences between the Display type (FLDs in dark-gray, PLDs in light-gray). In all the Panels, the boxes represent the interquartile range, the black horizontal lines represent the median, the black vertical bars represent the standard error. The black stars represent the significant differences (*p* < 0.05), the dashed line and gray star represent the marginally significant differences (0.05 < *p* < 0.01).

Results of the mixed ANCOVA showed that overall the accuracy significantly increased with Age and IQ [*F*_(__1_,_58__)_ = 12.576, *p* < 0.001, η^2^G = 0.091; *F*_(__1_,_58__)_ = 12.077, *p* < 0.001, η^2^G = 0.088, respectively]. There was a significant effect of Group [*F*_(__2,__58__)_ = 21.051; *p* < 0.001; η^2^G = 0.251], with TD children outperforming both the HFA (*p* < 0.001) and the LFA ones (*p* < 0.001), and the children with HFA outperforming the LFA ones (*p* < 0.001). Furthermore, a significant effect of Display [*F*_(__1_,_62__)_ = 11.211; *p* = 0.001; η^2^G = 0.013], with higher accuracy for FLDs compared to PLD; and a significant effect of Emotion [*F*_(__2_,_124__)_ = 6.228; *p* = 0.003; η^2^G = 0.036], with lower accuracy for happy stimuli than fearful (*p* = 0.001) and neutral (*p* = 0.001) stimuli, were found. The interaction between Display and Emotion was also significant [*F*_(__2_,_128__)_ = 3.216; *p* = 0.043; η^2^G = 0.005], with higher accuracy in FLDs than in PLDs only for happy stimuli (*p* = 0.004). Interestingly, also the interaction between Group and Emotion closely approached significance [*F*_(__4_,_124__)_ = 2.36; *p* = 0.057; η^2^_*G*_ = 0.027]. TD were more accurate than ASDs not only in recognizing emotional (happy and fearful) but also neutral stimuli, while differences between the two groups with ASD were found only in neutral stimuli (Table 2 and Figure 2A).

**TABLE 2:** The table shows the results of Bonferroni adjusted pairwise *t*-test in the three groups of children (HFA, LFA, and TD) in every emotional category.

	Post hoc comparison Emotion*Group		
	**Happiness**	**Fear**	**Neutral**
TD vs. HFA	*p* = 0.014	*p* = 0.044	*p* = 0.019
TD vs. LFA	*p* = 0.003	*p* < 0.001	*p* < 0.001
HFA vs. LFA	n.s	n.s.	*p* < 0.001

#### Analysis Within Single Groups

The high variability of accuracy in the ASD groups’ (see Figure 2 and Table 3 columns SD and SE) and the reduced sample size, especially in the LFA group, could have masked some effect, prevented some interactions to reach significance, and reduced the effect size. Therefore, to better understand the effect of Emotion and Display in the three groups of children, we performed three separated ANCOVAs with Emotion and Display as within-factors and IQ and Age as covariates (Figures 2B,C). The results showed a significant effect of Display only in TD children, with FLDs recognized more accurately than PLDs [*F*_(__1_,_24__)_ = 7.23, *p* = 0.013, η^2^G = 0.02]. The effect of Emotion resulted significant in TD children [*F*_(__2_,_48__)_ = 5.78, *p* = 0.006, η^2^G = 0.098], with happy stimuli recognized less accurately than fearful (*p* = 0.003) and neutral (*p* = 0.001) ones, while no difference between fearful and neutral stimuli emerged. The effect of Emotion tended toward significance also in children with ASD (HFA: *F*_(__2_,_44__)_ = 2.97, *p* = 0.062, η^2^G = 0.05; LFA: *F*_(__2_,_24__)_ = 3.14; *p* = 0.061, η^2^G = 0.04), therefore exploratory *post hoc* comparisons were performed also in these groups. Similarly to the TD group, in children with HFA the happy stimuli were recognized less accurately than neutral (*p* = 0.012) and fearful ones (*p* = 0.048), while fearful did not differ from neutral stimuli. Differently, in children with LFA the neutral stimuli were recognized with lower accuracy than fearful (*p* = 0.018) and marginally than happy ones (*p* = 0.056), while happy did not differ from fearful stimuli. The interaction between Display and Emotion was not significant in any of the group. Finally, the effects of Age [*F*_(__1_,_22__)_ = 8.66, *p* = 0.007, η^2^G = 0.156] and IQ [*F*_(__1_,_22__)_ = 7.18, *p* = 0.014, η^2^G = 0.133] resulted significant only in children with HFA.

**TABLE 3:** The table shows the mean, standard deviations (SD), and standard errors (SE) of Accuracy and RTs in each group (HFA, LFA, and TD) averaged by emotions (Fear, Happiness, and Neutral) and display types (FLDs and PLDs).

**Group**	**Emotion**	**Display**	**Accuracy (%)**			**RTs (msec)**		
			***Mean***	***SD***	***SE***	***Mean***	***SD***	***SE***
HFA	Fear	*FLDs*	81.18	19.31	4.55	1867.44	1438.07	338.96
	Fear	*PLDs*	77.80	21.02	4.95	1846.25	1474.54	347.55
	Happiness	*FLDs*	78.16	15.16	3.57	1956.79	1889.27	445.31
	Happiness	*PLDs*	75.20	16.49	3.89	2018.46	1655.59	390.23
	Neutral	*FLDs*	81.00	20.77	4.89	2058.36	2080.01	490.26
	Neutral	*PLDs*	83.20	16.70	3.94	1996.76	1508.31	355.51
LFA	Fear	*FLDs*	72.33	21.78	5.13	1709.65	1258.32	296.59
	Fear	*PLDs*	68.89	23.31	5.50	1920.32	1198.63	282.52
	Happiness	*FLDs*	75.00	20.35	4.80	1796.61	1144.20	269.69
	Happiness	*PLDs*	66.00	16.60	3.91	2018.21	1171.10	276.03
	Neutral	*FLDs*	66.00	20.63	4.86	1906.29	1183.49	278.95
	Neutral	*PLDs*	63.33	20.15	4.75	2028.01	1062.12	250.34
TD	Fear	*FLDs*	93.70	9.86	2.32	1199.03	315.38	74.34
	Fear	*PLDs*	89.26	10.80	2.55	1206.61	323.09	76.15
	Happiness	*FLDs*	89.26	8.63	2.03	1287.90	243.78	57.46
	Happiness	*PLDs*	83.89	13.33	3.14	1269.94	295.19	69.58
	Neutral	*FLDs*	94.07	7.08	1.67	1266.62	227.39	53.60
	Neutral	*PLDs*	92.78	10.32	2.43	1230.26	281.97	66.46

### Analysis of Response Times (RTs)

The results of the mixed ANCOVA showed that overall the RTs significantly decreased with Age and IQ [*F*_(__1_,_58__)_ = 4.83, *p* = 0.032, η^2^_*G*_ = 0.07; *F*_(__1_,_58__)_ = 8.803, *p* = 0.004, η^2^_*G*_ = 0.11, respectively]. The effect of Emotion was significant [*F*_(__2_,_124__)_ = 6.954; *p* = 0.001; η^2^_*G*_ = 0.008], with fearful stimuli recognized faster than happy (*p* < 0.001) and neutral ones (*p* = 0.003). The effect of Display closely approached significance [*F*_(__1_,_62__)_ = 3.806; *p* = 0.056; η^2^_*G*_ = 0.003], with faster RTs for FLDs than for PLDs. The effect of Group [*F*_(__2_,_58__)_ = 1.585; *p* = 0.214; η^2^_*G*_ = 0.043] and all the interactions were not significant (all *p*s > 0.05). Coherently with the analysis in Accuracy, we performed also three within-group ANCOVAs separately in the three groups, with Emotion and Display as within-factors and IQ and Age as covariates. Results showed a main effect of Emotion in TD children [*F*_(__2_,_48__)_ = 4.01, *p* = 0.024; η^2^_*G*_ = 0.03], with fearful stimuli recognized faster than happy ones (*p* = 0.002). The effect of Emotion was significant also in children with LFA [*F*_(__2_,_24__)_ = 3.78; *p* = 0.037; η^2^_*G*_ = 0.003], with fearful stimuli recognized faster than happy (*p* = 0.028) and neutral ones (*p* = 0.033). The effect of Display and the interaction between Display and Emotion were not significant in any of the group. Finally, the effects of Age and IQ were significant only in children with LFA [*F*_(__1_,_12__)_ = 6.64, *p* = 0.024, η^2^_*G*_ = 0.264; *F*_(__1_,_12__)_ = 18.17, *p* = 0.001, η^2^_*G*_ = 0.495, respectively].

#### Speed-Accuracy Trade-Off (SATO)

**FIGURE 3:**
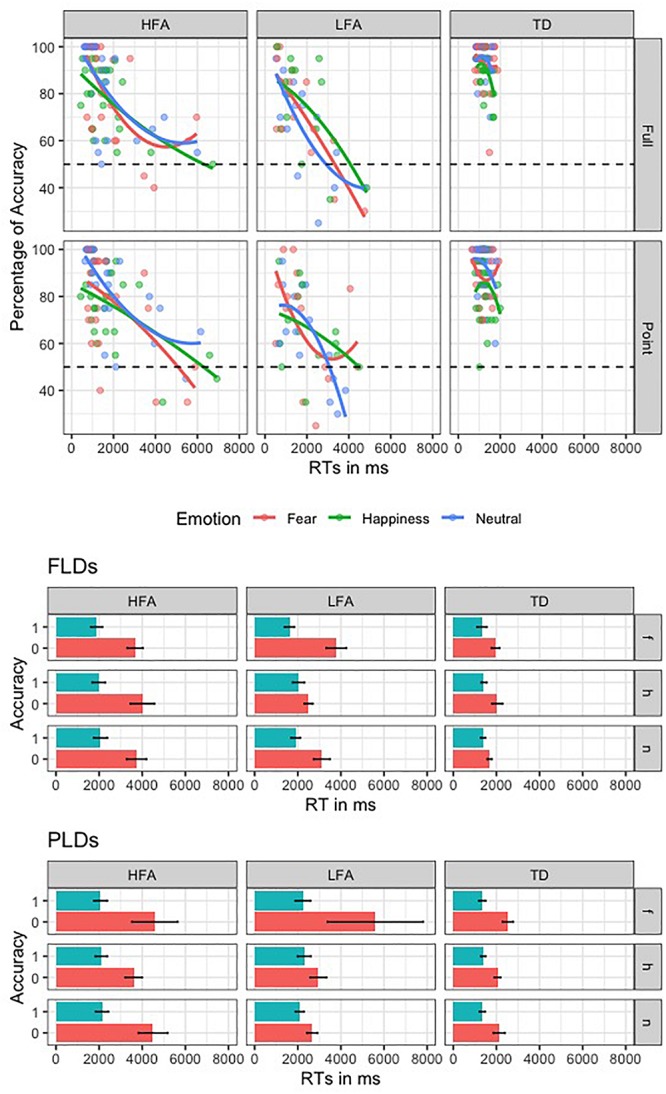
This figure shows the speed-accuracy trade-off in the three groups of children. In the first plot, the second order polynomial distribution of the RTs against Accuracy rate is represented in the three groups (columns) for the two display types (rows); lines and dots are colored according to the three emotions. The second and the third plots represent the mean RT of correct (blue) and incorrect (red) responses, relative to the three emotions (rows, “f” is for fear, “h” is for happiness, and “n” is for neutral) in the three groups (columns).

To investigate the SATO, we plotted the RTs in ms against the percentage of Accuracy for the three groups in recognizing the emotions with different Displays (Figure 3). Furthermore, we performed an ANOVA with logRT as dependent variable, Group as between-group factor (TD, HFA, and LFA), and Type of response (correct vs. incorrect) as within-group factor. The results showed a significant main effect of Group [*F*(2,62) = 5.80, *p* = 0.005 η^2^ = 0.115], with a greater logRT in HFA and LFA compared to TD [*p* = 0.001 and *p* = 0.005, respectively], but no differences between HFA and LFA. The main effect of Type of response was also significant [*F*(1,61) = 72.133, *p* < 0.001, η^2^ = 0.134], with greater logRT for incorrect vs. correct responses. Notably, the interaction between Group and Type of response was not significant, showing that in all the tree groups the incorrect answers corresponded to greater RTs. These results suggest that the greater number of errors in children with ASD are not due to a tendency to respond faster compared to TD ones.

## Discussion

We initially predicted that if the impairment in recognizing the emotional BM was due to a specific difficulty in emotion processing, the children with ASD should recognize the *emotional* stimuli with poorer accuracy and higher RTs, while they should not differ from TD children in recognizing the *neutral* stimuli. Our results showed that children with ASD, independently of their IQ level, were less accurate than TD children in recognizing both the emotional and the neutral BM, either when displayed as FLDs or PLDs. When the emotional content was correctly recognized, the RTs did not differ between the three groups. Additional analysis showed that the vision of the body form improved the recognition of BM only in TD children, while in children with ASD the accuracy did not differ between the two display types. According to our initial hypothesis, these results suggest that the impairment in recognizing the emotional content of BM in ASD is associated to the processing of BM information rather than being specific for the processing of the emotional content or the visual characteristics of the stimuli.

### Differences in Recognizing Emotional and Neutral BM

Previous studies about recognition of emotional BM in ASD found a specific difficulty in naming the emotional but not the neutral BM either in children and adults (Moore et al., 1997; Hubert et al., 2007; Parron et al., 2008). The discrepancies between our and existing results may be explained by methodological differences. For instance, in previous studies the participants were asked to verbally describe the observed PLDs using emotional words (Moore et al., 1997; Hubert et al., 2007; Parron et al., 2008). However, labeling the emotions may present more than a challenge to participants with ASD (Lartseva et al., 2014). Hence, it is possible that some language or memory difficulties (e.g., in knowing/recall/select the specific emotion label) have constrained the performance of individuals with ASD on the verbal emotion recognition tasks. For this reason, in the present study we tried to overcome the limitations of spontaneous verbal labeling of emotions by asking participants to perform a non-verbal two-forced-choice task. The forced-choice task reduces the cognitive load and helps to obviate the potential difficulties related to the recall/selection of the emotional label, which may have led to poorer performance in previous studies. This simplified non-verbal procedure, together with the non-verbal IQ as matching criteria, was crucial to allow also the participants with LFA to perform the emotion recognition task and to highlight differences and commonalities between children with ASD with and without ID.

Using the forced-choice task, we found that TD children were more accurate than children with ASD in recognizing both the emotional and the neutral stimuli. This shows that the attribution of an affective significance to BM is difficult for children with ASD, even when no verbal label is required, either when they have to recognize the presence (fearful and happy BM) or the absence (neutral BM) of emotions. Our results parallel the findings of two other studies that adopted a forced-choice task to investigate the emotional BM recognition in adults with ASD (Atkinson, 2009; Nackaerts et al., 2012) and supports the hypothesis that the difficulty in recognizing the affective meaning of BM is related to the processing of body motion – rather than emotion – information. This is not surprising, as in bodily expressions the emotional content is conveyed by the BMs, thus the correct recognition of the bodily-expressed emotion requires the correct encoding of the observed motion information (Chouchourelou et al., 2006; Roether et al., 2009). Nevertheless, this is consistent with the idea that abnormalities in processing high-order motor information can affect the social functioning in ASD and lead to difficulties in social domains (Oberman and Ramachandran, 2007; Rizzolatti and Fabbri-Destro, 2010; Casartelli et al., 2016). Evidence from neuroimaging studies seems to corroborate this hypothesis. At the brain level, the vision of bodily expressions activates a network of areas responsible for action understanding that comprises the superior temporal sulcus (pSTS) and the putative mirror-neuron system (MNS), together with visual, limbic and subcortical structures (Heberlein et al., 2004; Grèzes et al., 2007; de Gelder et al., 2010; Van den Stock et al., 2011). In particular, the MNS contains high-level motor representations that are active both during the execution and the observation of an action (for an extensive review, see Rizzolatti et al., 2014). Interestingly, transcranial magnetic stimulation (TMS) studies provided evidence that the perturbation of the BM-related areas alters the recognition of bodily expressions (Candidi et al., 2011; Engelen et al., 2015; Mazzoni et al., 2017), proving that these areas are causally involved in recognizing the affective meaning of BM. Consistently, fMRI findings showed abnormal activation of the neural network responsible for action understanding in individuals with ASD when presented with emotional BM (Freitag et al., 2008; Grèzes et al., 2009; McKay et al., 2012). Furthermore, results of an electromyographic study suggest that deficits related to the parietal node of MNS may underlie the difficulties in action comprehension in children with ASD (Cattaneo et al., 2007). This body of evidence suggests that structural, functional, and connectivity abnormalities in the action-related areas in ASD may prevent the correct encoding of the observed BM. In turn, this may produce difficulties in recognizing the observed BM, both emotional and neutral, that is indeed what we found in the present study.

### Differences Between Emotions

We found that the three emotional types were recognized with different accuracy and different RTs in the three groups: in children with HFA, the effect of Emotions was significant in accuracy, with TD-like poorer accuracy in happy stimuli compared to fearful and neutral ones, but it was not significant in RTs; in children with LFA, the recognition of the neutral BM was accomplished with the poorest accuracy, beside they showed TD-like faster RTs for fearful stimuli. Notwithstanding the nature of the additional within-group analysis was exploratory and should be interpreted with caution, these results highlight the importance of including both the children with HFA and LFA in research aimed at investigating the recognition of emotional BM in ASD and to consider them as separate groups.

Notably, in all the three groups there was an advantage for the fearful stimuli. This is in line with other behavioral studies on recognition of emotional BM (Atkinson et al., 2004; Bannerman et al., 2009; Philip et al., 2010). When a body expression is perceived, the priority in our brain is to create a representation of the observed emotional movements in order to react promptly and adaptively (Grèzes et al., 2007). In particular, fear communicates the presence of potential threats in the environment and it is associated with increased vigilance and attention (Phelps et al., 2006; Tamietto et al., 2007; Kret et al., 2013), enhanced visual processing (van Heijnsbergen et al., 2007; Borhani et al., 2015), and modulation of the motor system (Borgomaneri et al., 2012, 2015). Our results are in line with these studies and showed that fearful BM was recognized more accurately in TD children and in children with HFA, and its recognition was faster in TD children and in children with LFA, compared to the recognition of happy or neutral stimuli.

Another interesting result is that the children with LFA recognized with poorest accuracy the neutral stimuli. Our hypothesis it that this could be due to the fact that the recognition of emotional states is reinforced in social interaction, whilst the recognition of “neutral” state is not. Indeed, since very early in development, the emotions are highlighted and reinforced in natural social exchange. For instance, the parents categorize the emotions and the internal states of the child while she/he is experiencing them, using the emotional label corresponding to the facial or bodily expressions (e.g., “You play with the ball! Oh look at you, you are smiling, you are happy! You like it!”). This reinforces the coupling of emotional non-verbal stimuli (such as facial and bodily expressions) with the concurrent action and internal state, and serves the acquisition of the ability to recognize the emotional expressions. Differently, it is rare that this reinforcement is done with the neutral expressions, neither during daily social interaction, nor during training or intervention with children with ASD. Indeed, when the attention is focused on the neutral actions, it is more probable reinforced the type of action and its functional significance, rather than its non-emotional content. As a consequence, children with ASD can create internal representations of the emotional expressions, but not of the neutral ones. Nevertheless, it is possible that the children with HFA, thanks to the higher IQ, can learn to associate the neutral expressions with the absence of any emotions. However, if the IQ level is poor, as it is in individuals with LFA, this explicit strategies will be learned with difficulty or not learned at all. This might be the reason why the recognition of the neutral stimuli was particularly difficult in our group of children with LFA.

### Differences Between PLDs and FLDs

Most of the previous studies have investigated the BM recognition in ASD using only PLDs. However, in the real life the body form is fully visible and the use of realistic stimuli should hence be considered – if not encouraged – in research aimed at explaining the origin of daily social difficulty in clinical populations. Indeed, to ascribe the social impairment in ASD to deficit in processing the BM information, the difficulty should be consistent independently of the visual representation of BM (e.g., pure kinematic or fully visible body form). The present work contributes to the small number of studies that have investigated the emotional BM recognition using both FLDs and PLDs. So far, this evidence in children with ASD has been missed. Our results showed a difficulty in children with ASD, compared to TD children, in recognizing the emotional BM when presented as PLDs (i.e., simplified stimuli). This is consistent with previous studies with PLDs (Moore et al., 1997; Hubert et al., 2007; Parron et al., 2008; Nackaerts et al., 2012) and confirm a difficulty in children with ASD in understanding the significance of BM represented as PLDs. In addition, we strikingly found that the accuracy in children with ASD was poorer than that of TD children also in recognizing the FLDs. According to our initial predictions, this result supports the hypothesis that the impaired recognition of BM in ASD is related to higher-order motion processing, which underlie the encoding of BM independently of its visual representation.

Furthermore, within group analysis showed that TD’s accuracy was higher when the body FLDs, while in children with ASD no difference between PLDs and FLDs emerged. According to the models for BM processing (e.g., Giese and Poggio, 2003), the BM identification requires the integration of form and motion information, likely associated with the activation and involvement of the superior temporal sulcus (pSTS). Specifically, the authors proposed a hierarchical model for BM processing with two pathways, one for form and the other one for motion information. In the model, the form pathway goes from visual cortex to inferior temporal cortex, the fusiform cortex, and STS. The motion pathway goes from visual cortex to MT and subsequently to STS and fusiform cortex. The two pathways merge into STS that integrate information from both streams. From STS, the BM information are then projected to the frontal and parietal areas. The posterior part of STS is indeed part of the action observation network and provide the main visual input to the fronto-parietal regions of the putative mirror neuron system (namely, the inferior frontal gyrus and the inferior parietal lobule) (for an exernsive review, see Rizzolatti et al., 2014). Our results suggest that this integration of form and motion information occurs, in TD children and facilitates the recognition of the emotional content of BM, while it seems to be impaired in children with ASD. Consistently, neuroimaging findings in ASD have shown structural abnormalities in STS (McAlonan et al., 2005; Zilbovicius et al., 2006; Courchesne et al., 2007); atypical activation in STS during BM perception (Freitag et al., 2008; Kaiser et al., 2010; McKay et al., 2012); and significant reduced connectivity between STS and the fronto-parietal areas processing higher-order motion information (McKay et al., 2012; Alaerts et al., 2014). Hence, we hypothesize that these abnormalities in STS and in its connections with the motor areas of the MNS may result in a lack of integration of form and motion cues, which in turn may lead to the impaired comprehension of BM and, thus, may explain the different recognition of FLDs and PLDs that we found between the children with and without ASD.

### Relation Between IQ, Age, and the Recognition of Body Movements

As predicted, results of the overall analysis showed that the accuracy and speed in recognizing the emotional BM improved with age and IQ. This finding matches another study on basic and complex emotion recognition (Fridenson-hayo et al., 2016). In this study, the FLDs were employed in 5–9 years old children with TD and HFA, a significant relation with this ability and the age was found.

In children with ASD, we found that the effect of IQ and Age were significant only in children with HFA for what concerns the accuracy, and only in children with LFA for what concerns the RTs. These results suggested that higher cognitive resources in children with HFA might subtend the acquisition of alternative strategies useful for BM recognition (Rutherford and Troje, 2012), which proficiency seems to improve with age. It is plausible, in fact, that when the mechanism for BM perception is altered, as it is in children with ASD (Kaiser et al., 2010; Kaiser and Pelphrey, 2012; Mcpartland et al., 2012), the recognition of emotional BMs it is likely mediated by alternative strategies (McKay et al., 2012). For instance, using a point-light direction discrimination paradigm, McKay et al. (2012) found a comparable behavioral performance, but different brain activation, in adults with and without ASD. This suggests that individuals with ASD may accomplish the same task using different brain regions compared to TD participants, yet reaching similar proficiency levels. In particular, in TD group a unitary network of areas, which included temporal and parietal regions, was found to be active. On the contrary, this activation was missing in the ASD group and two other distinct networks were activated, involving brain areas selective for motion and form encoding, respectively. In other words, in people with ASD there seems to be a lack of implicit and automatic process of emotional BM. To compensate this lack, individuals with high level of IQ may develop explicit strategies that serve the emotional BM recognition by recruiting cognitive processes that are specialized for the encoding of non-social stimuli. Presumably, these strategies are declarative and learned explicitly through experience and/or training, they are sophisticated and require considerable cognitive efforts. Therefore, they can be developed only in presence of high cognitive resources. Our results are consistent with these findings and, in addition, we found that this alternative and compensatory strategies improved with age through childhood and, potentially, would reach TD-like performance later in life. Future studies that investigate the differences in performance between individuals with HFA at different ages (e.g., childhood, adolescence, and adulthood) and age matched control groups would help better understanding this developmental trend.

With regard to the children with LFA, we found that the accuracy was not predicted neither by the age nor by the IQ, but these effects resulted significant in RTs analysis. This suggests that, even though the difficulties of children with LFA in understanding the emotional meaning of BM remain stable during the development, when they are successful in comprehending the BM they do it more rapidly with age, and the rapidity is increased by higher non-verbal IQ.

In TD children we found that this ability was not modulated by age and IQ, nor for the accuracy neither for the RTs. This result is partially contrary to our initial hypothesis and to previous findings in children with TD (Ross et al., 2012). However, this incongruence may result from the narrower range of age and the limited sample size of our study, which could have masked some effects. Indeed, whereas our study involved 27 TD children between the age of 5 and 11 years, the study of Ross and collaborators included a larger sample of 107 children aged between 4 and 17 years old. Another possible explanation could be ascribed to the type of task: our task had a reduced load of working memory with respect to that of Ross and collaborators, as it was aimed at testing children with LFA, therefore it could have been less sensible to detect age-related changes in TD.

A few limitations of the study should be noted: the reduced sample size and the high variability of responses, especially in children with LFA, could have prevented the interaction between group and displays and between group and emotion to reach significance in the overall analysis of accuracy and RTs. Whereas some effects clearly emerged from the additional within-group analysis, the nature of these analysis is exploratory and, therefore, the data should be interpreted with caution. For these reasons, further studies with larger sample size are needed before firm conclusions can be drawn. Despite these limitations, our findings contribute to the very small number of studies on emotional BM recognition that have involved people with LFA and could be the starting point for future research aimed to better understand this ability in subgroups of people with ASD with different characteristics. To better understand differences and commonalities between individuals LFA and HFA, future investigation of emotional BM recognition in ASD should also be extended to other emotional contents (e.g., including anger and sadness), comparisons between different sources of social and emotional stimuli (e.g., faces, bodies, and voices). Finally, different range of age and longitudinal studies are desirable to examine the developmental trajectories of BM recognition in ASD.

## Data Availability Statement

The datasets generated for this study are available on request to the corresponding author.

## Ethics Statement

The studies involving human participants were reviewed and approved by the Ethical Committee of the University of Milano-Bicocca. Written informed consent to participate in this study was provided by the participants’ legal guardian/next of kin.

## Author Contributions

NM, PR, RA-G, and PV discussed the original idea of the project and designed the study. NM collected the data, performed the statistical analysis, and drafted the manuscript. IL performed the statistical analysis and edited the manuscript. PV, PR, and RA-G edited the manuscript. All authors revised the article.

## Conflict of Interest

The authors declare that the research was conducted in the absence of any commercial or financial relationships that could be construed as a potential conflict of interest.

## Abbreviations

ASD: autism spectrum disorder
BM: body movement
FLDs: full-light display stimuli
HFA: high-functioning autism spectrum disorder
ID: intellectual disability
LFA: low-functioning autism spectrum disorder
PLDs: point-light display stimuli
TD: typical developing.
